# Epidemiological characteristics in serotype 24 paediatric invasive pneumococcal disease according to an 11-year population-based study in Japan

**DOI:** 10.1017/S0950268822000395

**Published:** 2022-02-28

**Authors:** Kenichi Takeshita, Noriko Takeuchi, Misako Ohkusu, Haruka Hishiki, Yuki Shiko, Yohei Kawasaki, Bin Chang, Naruhiko Ishiwada

**Affiliations:** 1 Department of Pediatrics, Graduate School of Medicine, Chiba University, 1-8-1 Inohana, Chuo-ku, Chiba-shi, Chiba, Japan; 2 Department of Infectious Diseases, Medical Mycology Research Center, Chiba University, 1-8-1 Inohana, Chuo-ku, Chiba-shi, Chiba, Japan; 3 Biostatistics Section, Clinical Research Center, Chiba University Hospital, 1-8-1 Inohana, Chuo-ku, Chiba-shi, Chiba, Japan; 4 Faculty of Nursing, Japanese Red Cross College of Nursing, 4-1-3 Hiroo, Shibuya-ku, Tokyo, Japan; 5 Department of Bacteriology I, National Institute of Infectious Diseases, 1-23-1, Toyama, Shinjuku-ku, Tokyo, Japan

**Keywords:** Invasive pneumococcal disease, serotype 24, 13-valent pneumococcal conjugate vaccine, serotype replacement

## Abstract

After the introduction of the 13-valent pneumococcal conjugate vaccine (PCV13), serotype replacement has occurred in Japan, and serotype 24 has become the most common serotype in paediatric invasive pneumococcal disease (IPD). To understand the characteristics of serotype 24-IPD in Japanese children in the post-PCV13 era, we conducted a retrospective study in children aged ≤15 years from 2010 to 2020 using a database of paediatric IPD surveillance in Chiba prefecture, Japan. We identified a total of 357 IPD cases and collected clinical information on 225 cases (24: 32 cases, non-24: 193 cases). Compared with the non-serotype 24-IPD, serotype 24-IPD was independently related to be <2 years of age [odds ratio (OR) 3.91, 95% confidence interval (CI) 1.47–10.44; *P* = 0.0064] and bacteremia (OR 2.28, 95% CI 1.01–5.13; *P* = 0.0475), as a result of the multivariate regression analysis. We also conducted a bacterial analysis, and the isolates of serotype 24-IPD had tendencies of PCG-susceptible (24: 100.0%, non-24: 61.3%; *P* < 0.0001) and macrolide-resistance (24: 100.0%, non-24: 87.3%; *P* = 0.0490). Their multilocus sequence typing was mostly ST2572 and the variants, which were unique to Japan. This tendency might have been a result of the progress made in the Japanese PCV13 immunisation programme.

## Introduction

*Streptococcus pneumoniae* is a major pathogen that causes not only respiratory diseases such as pneumonia and acute otitis media but also life-threatening diseases such as meningitis and bacteremia; thus, it is referred to as invasive pneumococcal disease (IPD). Pneumococcal conjugate vaccine (PCV) was introduced for universal use in children in many countries, including Japan, and the incidence of IPD in children was subsequently remarkably decreased; however, non-PCV serotype-IPD has increased, so-called ‘serotype replacement’, which is a difficult problem in the world [1].

In Japan, a 13-valent pneumococcal conjugate vaccine (PCV13) was introduced in November 2013 to replace the heptavalent pneumococcal conjugate vaccine (PCV7) for universal use in children aged ≤5 years. Since the introduction of PCV13, more than 95% of children have routinely received this vaccine, and there has been an acceleration in serotype replacement [2, 3]. In particular, serotype 24, a type of non-PCV13 serotype, is rapidly increasing, and, consequently, this has become the most common serotype in paediatric IPD in 2015 (from 2015 to 2019) according to a Japanese nationwide active population-based surveillance in ten prefectures [4]. Notably, this serotype is not so common in Japanese adults [5]. In some countries worldwide, the prevalence of serotype 24-IPD is increasing in children; however, this is not increasing in some of the other countries [6].

It is important to clarify the characteristics of serotype 24-IPD which is increasing in Japanese children in the post-PCV13 era, to enable progress in the strategy of prevention and treatment against pneumococcal infection. We have been conducting an ongoing epidemiological survey regarding paediatric IPD in a Japanese prefecture before the introduction of PCV. Using data from this survey, we conducted an 11-year retrospective study to determine the epidemiological and clinical features of serotype 24-IPD in Japanese children.

## Methods

### Study setting and design

We retrospectively collected information about IPD cases involving people aged ≤15 years from January 2010 to December 2020 from a surveillance database of paediatric IPD in Chiba prefecture. This prefecture is located east of Tokyo and has a population of 6.3 million people, 757 000 of whom were aged ≤15 years in 2020 [7]. This surveillance was a population-based study based on the report from all the hospitals in which paediatric patients with IPD were treated in this prefecture.

During our surveillance, we also collected and investigated *S. pneumoniae* isolates from IPD cases. Collected isolates were primarily analysed for serotyping, antimicrobial susceptibility, and multilocus sequence typing (MLST) at the Medical Mycology Research Center, Chiba University, and then sent to the National Institute of Infectious Diseases to confirm the results.

We investigated the clinical epidemiology of paediatric IPD in serotype 24 cases and in non-24 cases, excluding cases that lacked serotype data. We also investigated the bacterial epidemiology, antimicrobial susceptibility, and MLST of collected isolates by serotype 24 and non-24 from the data of recorded cases.

### Bacterial analysis

We performed an analysis based on methods that were previously described [8, 9]. Each isolate was identified as *S. pneumoniae* using an optochin susceptibility test, and polymerase chain reaction (PCR) assays targeting the *lytA* gene, which encodes the major pneumococcal autolysin (*LytA*), were also used to identify *S. pneumoniae*. Serotyping was mainly performed using the Quellung reaction with pneumococcal antisera (Statens Serum Institut, Copenhagen, Denmark) and partly performed by a latex agglutination method or PCR method. Antimicrobial susceptibilities to penicillin G (PCG), cefotaxime (CTX), meropenem (MEPM), erythromycin (EM), clindamycin (CLDM), tosufloxacin (TFLX) and vancomycin (VCM) were analysed using the broth microdilution method according to the Clinical and Laboratory Standards Institute (CLSI) M07-A11 protocol. The minimum inhibitory concentration (MIC) breakpoints were defined according to the CLSI criteria (CLSI M100-30). We performed MLST as described previously [10]. Sequence types (STs) were determined by comparing the derived sequences of each locus to all known alleles by referencing the MLST database [11].

### Ethics statement

This study was approved by the Chiba University Ethics Committee No. 666 and conducted according to the principles expressed in the Declaration of Helsinki.

### Statistical analysis

Statistical analyses were performed using the JMP 15 (SAS Institute, Cary, NC, USA). Between-group differences in patient characteristics were analysed using Fisher's exact tests. We evaluated the comparison of antimicrobial susceptibility between serotype 24F and non-24F by break point using Fisher's exact tests. Univariate and multivariate regression analyses of clinical variables probably related to serotype 24F presumed by Fisher's exact tests were performed using logistic regression and the odds ratio (OR) and associated 95% confidence interval (CI) were calculated. All *P* values represented two-tailed tests, with *P* < 0.05 considered to be statistically significant.

## Results

### Study population

**Fig. 1.**
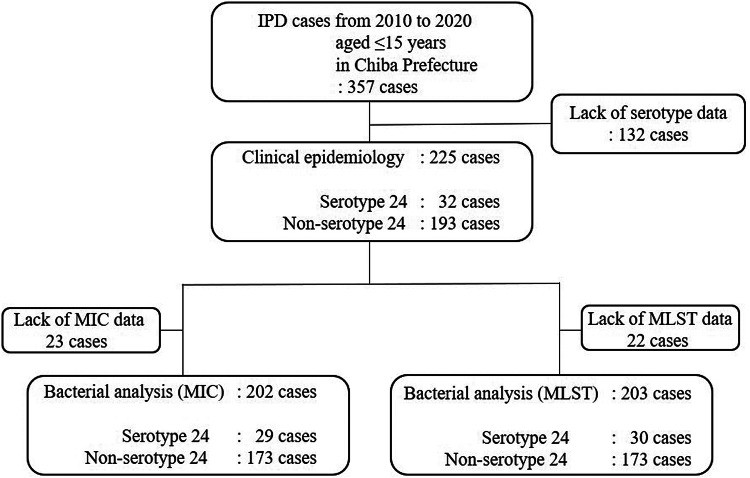
Flowchart of the retrospective study design. IPD, Invasive pneumococcal disease; MIC, minimum inhibitory concentration; MLST, multilocus sequencing typing.

Figure 1 is the flow chart of this study. We identified a total of 357 IPD cases from 23 paediatric facilities during the study period, of which 132 cases were excluded because of a lack of serotype information.

### Serotype distribution

Supplementary Fig. S1 displays the serotype distribution of 225 IPD cases. The percentage of PCV13 serotypes among all identified serotypes was 73.1% during 2010–2012; however, from 2013 to 2020, this proportion decreased to 17.1%, and the percentage of non-PCV13 serotypes increased to 82.9%, which can be considered to be serotype replacement. Serotype 24 was found in 32 of the 225 cases (serotype 24F: 25 cases, 24B: 6 cases, and unknown subtype: 1 case) which accounted for 14.2% of all identified. The proportion of this serotype-IPD increased after introducing PCV13 and became the highest in the post-PCV13 era (2010–2012: 4.5%, 2013–2020: 18.4%).

### Clinical analysis of 24-IPD cases as compared with non-24-IPD cases

Table 1 presents the clinical characteristics of 225 IPD cases according to 24 and non-24 cases. We compared 32 cases of 24-IPD and 193 cases of non-24-IPD. The number of 24-IPD cases was significantly higher from 2013 to 2020, the post-PCV13 era (24: 90.6%, non-24: 66.8%; *P* = 0.0060). As compared with the non-24-IPD cases, 24-IPD cases had a tendency to be aged <2 years (24: 81.3%, non-24: 52.9%; *P* = 0.0033), to have a history of PCV13 vaccination (24: 71.9%, non-24: 40.1%; *P* = 0.0010), and to have a history of bacteremia (24: 62.5%, non-24: 40.4%; *P* = 0.0219). No significant difference was found between the two groups in the proportion of sex, underlying diseases, presence of siblings, day care attendance, and outcomes.

**Table 1.** Clinical characteristics

Characteristic	Number (%) of patients			
	Total *n* = 225	24 *n* = 32	non-24 *n* = 193	*P*-value
Year of admission				
2000–2012	67 (29.8)	3 (9.4)	64 (33.2)	0.0060
2013–2020	158 (70.2)	29 (90.6)	129 (66.8)	
Age group, year				
<2	128 (56.9)	26 (81.3)	102 (52.9)	0.0033
2–5	74 (32.9)	6 (18.7)	68 (35.2)	
>5	23 (10.2)	0 (0.0)	23 (11.9)	
Male	113 (50.2)	13 (40.6)	100 (51.8)	0.2582
Underlying diseases^a^	54 (24.1)	4 (12.5)	50 (26.0)	0.1196
Presence of siblings^a^	91 (46.4)	13 (43.3)	78 (47.0)	0.8428
Daycare attendance^a^	122 (61.9)	18 (60.0)	104 (62.3)	0.8400
Vaccination history^a^				
PCV13	100 (44.6)	23 (71.9)	77 (40.1)	0.0010
PCV7	50 (22.4)	6 (18.8)	44 (23.0)	0.8189
PPSV23	1 (0.44)	0 (0.0)	1 (0.5)	1.0000
None	72 (32.3)	3 (9.4)	69 (36.1)	0.0020
Clinical presentations				
Bacteremia (no other symptom)	98 (43.6)	20 (62.5)	78 (40.4)	0.0219
Meningitis	30 (13.3)	1 (3.1)	29 (15.0)	0.0895
Becteremic pneumonia	48 (21.3)	6 (18.8)	42 (21.8)	0.8185
Bacteremia + respiratory infection	81 (36.0)	9 (28.1)	72 (37.3)	0.4267
Others	20 (8.9)	2 (6.3)	18 (9.3)	0.7465
Outcome^a^				
Sequelae	6 (3.8)	0 (0.0)	6 (4.4)	1.0000
Deaths	3 (1.9)	0 (0.0)	3 (2.2)	1.0000

PCV7, heptavalent pneumococcal conjugate vaccine; PCV13, 13-valent pneumococcal conjugate vaccine; PPSV23, 23-valent pneumococcal polysaccharide vaccine.

a Data were not fully collected.

To confirm the relativities to serotype 24, we carried out a multivariate regression analysis for four probable related factors (admission in 2013–2020, <2 years of age, a history of PCV13 vaccination, and bacteremia). As a result of this analysis, serotype 24-IPD was positively related to be <2 years of age (OR 3.91, 95% CI 1.47–10.44; *P* = 0.0064) and bacteremia (OR 2.28, 95% CI 1.01–5.13; *P* = 0.0475), independently, as presented in Table 2.

**Table 2.** Univariate and multivariate regression analysis for probable factors related to serotype 24

	Univariate			Multivariate		
	OR	95% CI	*P*-value	OR	95% CI	*P*-value
Age group <2 years	3.87	1.52–9.81	0.0044	3.91	1.47–10.44	0.0064
Bacteremia (no other symptom)	2.46	1.14–5.31	0.0223	2.28	1.01–5.13	0.0475
Year of admission 2013–2020	4.80	1.41–16.3	0.0122	3.89	0.88–17.17	0.0732
Vaccination history PCV13+	3.82	1.68–8.69	0.0014	1.51	0.54–4.24	0.4343

OR, odds ratio; CI, confidence interval; PCV, pneumococcal conjugate vaccine.

### Bacterial analysis of serotype 24 *S. pneumoniae* strains as compared with non-serotype 24 strains

Twenty-three cases that lacked MIC information were excluded from the 225 cases. Table 3 presents the comparison of antimicrobial susceptibility in serotype 24 (29 strains) with non-24 (173 strains) isolates from study patients. The proportion of PCG-sensitive isolates in serotype 24 strains with a susceptibility break point of meningitis (MIC ≤ 0.06) was significantly higher than that in non-serotype 24 strains (24: 100.0%, non-24: 61.3%; *P* < 0.0001). Moreover, the proportion of macrolide-resistance isolates in serotype 24 strains was significantly higher than that in non-serotype 24 strains (EM; 24: 100%, non-24: 87.3%; *P* = 0.0049, CLDM; 24: 100%, and non-24: 67.1%; *P* < 0.0001). We tested MLST for 30 strains of serotype 24 and found four STs, as displayed in Table 4. The most prevalent ST in serotype 24F was ST2572, which was also the most prevalent in serotype 24B. The second most prevalent ST in serotype 24F was ST5496, which was a single-locus variant of ST2572.

**Table 3.** Comparison of antimicrobial susceptibility in serotype 24 with non-24 *S. pneumoniae* isolates from study patients

		24			Non-24			
Antimicrobial agent	Susceptibility break points	MIC50	MIC range	% of S	MIC50	MIC range	% of S	*P*-value^a^
PCG	MIC ≤ 0.06	≤0.015	≤0.015–0.06	100.0	0.06	≤0.015–2	61.3	<0.0001
CTX	MIC ≤ 0.5	≤0.03	≤0.03–0.12	100.0	0.12	≤0.03–2	89.6	0.0815
MEPM	MIC ≤ 0.25	0.015	≤0.008–0.015	100.0	0.015	≤0.008–1	90.2	0.1396
EM	MIC ≤ 0.25	>8	2–8<	0.0	>8	≤0.12–8<	12.7	0.0490
CLDM	MIC ≤ 0.25	>8	0.5–8<	0.0	>8	≤0.12–8<	32.9	<0.0001
TFLX^b^	MIC ≤ 0.25	0.25	≤0.12–0.25	100.0	0.25	≤0.12–0.5	97.5	1
VCM	MIC ≤ 1	0.25	0.25–0.5	100.0	0.25	≤0.12–0.5	100.0	1

MIC, minimum inhibitory concentration; S, susceptibility; PCG, penicillin G; CTX, cefotaxime; MEPM, meropenem; EM, erythromycin; CLDM, clindamycin; TFLX, tosufloxacin; VCM, vancomycin.

a The results of the comparison of the percentage of susceptibility in 24 with non-24 isolates.

b Data from 145 patients (24F: 26, non-24F: 119).

**Table 4.** Varieties of multilocus sequence typing in serotype 24 *S. pneumoniae* isolates from study patients

Serotype	ST	*aroE*	*Gdh*	*gki*	*recP*	*spi*	*xpt*	*ddl*	Number of isolates
24F	2572	7	75	9	6	25	6	14	13
	5496	7	257	9	6	25	6	14	10
	162	7	11	10	1	6	8	14	1
	New	7	75	9	6	25	6	6	1
24B	2572	7	75	9	6	25	6	14	4
	2754	2	8	70	16	6	19	18	1

ST, sequence type.

## Discussion

We conducted an epidemiological study of paediatric IPD in Japan, focusing on serotype 24. We found that serotype 24-IPD increased after the introduction of PCV13, and this serotype was found significantly more often in the case of bacteremia in children who were <2 years old. The isolates of serotype 24-IPD had tendencies of PCG-susceptible and macrolide-resistance, and their STs were mostly ST2572 and the variants. This is the first report on the clinical and bacterial characteristics of serotype 24 in Japan based on statistical analyses.

PCV7 was first introduced in Japan in February 2010. The approved immunisation schedule has been described as a 3 + 1 schedule, consisting of three doses for the primary series and one booster injection. PCV7 was introduced in April 2013 for universal use in children aged ≤5 years and was replaced in November 2013 by PCV13. A previous epidemiological study in Japan demonstrated that the prevalence of non-PCV13 serotype isolates, especially that of serotype 15 strains and 24 strains, increased significantly from 2012 to 2014 in IPD patients between the ages of 2 months and 15 years, whereas serotype 24 did not increase in non-IPD patients [2]. Another study demonstrated that nine serotypes (10A, 12F, 15A, 15B, 15C, 22F, 24F, 33F and 35B) increased in paediatric IPD patients after the introduction of PCV7 and PCV13. In this study, serotype 24F greatly increased in paediatric IPD patients and slightly increased in adult IPD patients as well, that might be due to the influence of transmission from children to adults [3].

The prevalence of serotype 24 differs between countries. In a systematic review of serotype distribution in paediatric IPD (for young children under 5 years old) in the post-PCV era, the authors reported that 24F appeared to be prevalent in Europe and Western Pacific regions but not in North America [6]. In Europe, the prevalence of serotype 24F isolates increased in children after the introduction of PCV13 [12–15]. The IPD surveillance report of the European Centre for Disease Prevention and Control in 2017 indicated that serotype 24F was the most frequent serotype in IPD patients aged 1–4 years [16]. The authors of a study in France investigated the serotype of isolates from paediatric IPD patients and carriage isolates from healthy children and found that only serotype 24F was associated with IPD in children aged 6 to 24 months in the post-PCV13 era [17]. The major disease type of IPD by 24F differs among countries. For example, meningitis by serotype 24F is common in France; however, non-meningitis-like bacteremia is common in Germany [13, 14].

The results of our study indicate that serotype 24 was associated with IPD in young ages, especially <2 years of age, in the post-PCV13 era, which is similar to the epidemiology in Europe. Moreover, we found that bacteremia was a common type of IPD by serotype 24, which is the same as that found in Germany. Thus, the clinical epidemiology of serotype 24-IPD in Japan is similar to that in other countries, where PCV13 had been introduced.

In the bacterial analysis, we found that all serotype 24 isolates were susceptible to PCG. This characteristic is similar to that reported in a previous study in Japan [2] but different from that in a study conducted in Europe. In Europe, some serotype 24F isolates were found to be non-susceptible to PCG, and the report of a multidrug-resistant strain was also found [17, 18]. Identified MLSTs were also different from those identified in Europe. In our study, mostly ST2572 and the variants were identified in serotype 24F isolates; however, the major STs of serotype 24F in Europe were ST72 and ST162, as reported from Denmark [19]. According to the public database of MLST, in addition to these STs, ST177 and ST230 were often identified in serotype 24F from the United Kingdom and France [11]. Interestingly, ST2572 strains in this study were identified not only in serotype 24F isolates but also in serotype 24B isolates, and 24B-ST2572 strain have not been reported before except in Japan. Capsular switching might have occurred between 24F and 24B. Regarding the other STs we identified, it was notable that ST162 isolate was identified in serotype 24. This sequence type is known for capnophilic strain and it has not been reported in serotype 24 in Asia [11, 20]. In terms of bacterial epidemiology, the increase in the prevalence of serotype 24-IPD in young children in Japan might be caused by the original strain, which differs from the strain in other countries.

Considering our clinical and bacterial investigations, we propose two hypotheses for the increase of the serotype 24-IPD in children who were <2 years old during the post-PCV13 era. The first hypothesis is that it is due to the strong suppression of pneumococcal carriage and infection in PCV13 serotypes. In Japan, vaccine coverage for PCV13 is very high, and more than 95% of children have completed the 3 + 1 routine vaccinations by 2 years of age [21]. Just as with serotype replacement on IPD isolates, the distribution of serotype in the nasopharyngeal carriage in healthy children who are 2 years of age has also changed, and the carriage rate of the PCV13 serotype has decreased [22]. In our study, 73.1% of 24-IPD children aged <2 years were 1 years old, and most of them had received PCV13. We suspect that the strong suppression of the PCV13 serotypes carriage made it easier for the serotype 24 strain to colonise the nasopharynx in these patients and cause IPD. The second hypothesis is the highly invasiveness of serotype 24 in the Japanese strain. In young children, the mucosal barrier in the nasopharynx is immature, and a highly invasive strain can easily break through it, resulting in bacteremia without colonisation. The capsular serotype is a major determinant of the duration of carriage and invasiveness [23]; for example, serotype 12F is highly invasive, and bacteremia occurs easily without colonisation [24]. Because the colonisation of serotype 24 is rare in Japanese children [22, 25], we suspect that this strain was highly invasive and caused IPD, most of which was bacteremia without colonisation, as described in a previous case report [26].

This study has some limitations. First, this study is not nationwide surveillance and we did not consider the regional differences in Japan; however, this population-based study covered all <15-year-old-people in this prefecture, which was 5% in Japan. Second, we did not obtain carriage data of IPD patients and did not investigate their serotype-specific immunities to IPD serotype and vaccine serotypes. Finally, we did not analyse invasive factors such as pneumolysin of isolated strains.

In conclusion, we conducted an 11-year retrospective study regarding the serotype 24-IPD in children and found that serotype 24-IPD increased after the introduction of PCV13, and this serotype was found significantly more often in the case of bacteremia in children who were <2 years old. All isolated serotype 24 strains were PCG-susceptible and macrolide-resistance, and their MLSTs were ST2572, which is a unique strain in Japan, and its variants. This tendency might be the result of the progress made in the Japanese PCV13 immunisation programme. Further clinical and bacterial studies are required to clarify the mechanism of increasing serotype 24-IPD in children in the post-PCV13 era.

## Supplementary material

For supplementary material accompanying this paper visit http://doi.org/10.1017/S0950268822000395. click here to view supplementary material
