# Supplementary figures and images for: Epidemiological characteristics in serotype 24 paediatric invasive pneumococcal disease according to an 11-year population-based study in Japan
Source: Epidemiol Infect. 2022 Feb 28;150:e66. doi: 10.1017/S0950268822000395 (PMC8950979; doi:10.1017/S0950268822000395)

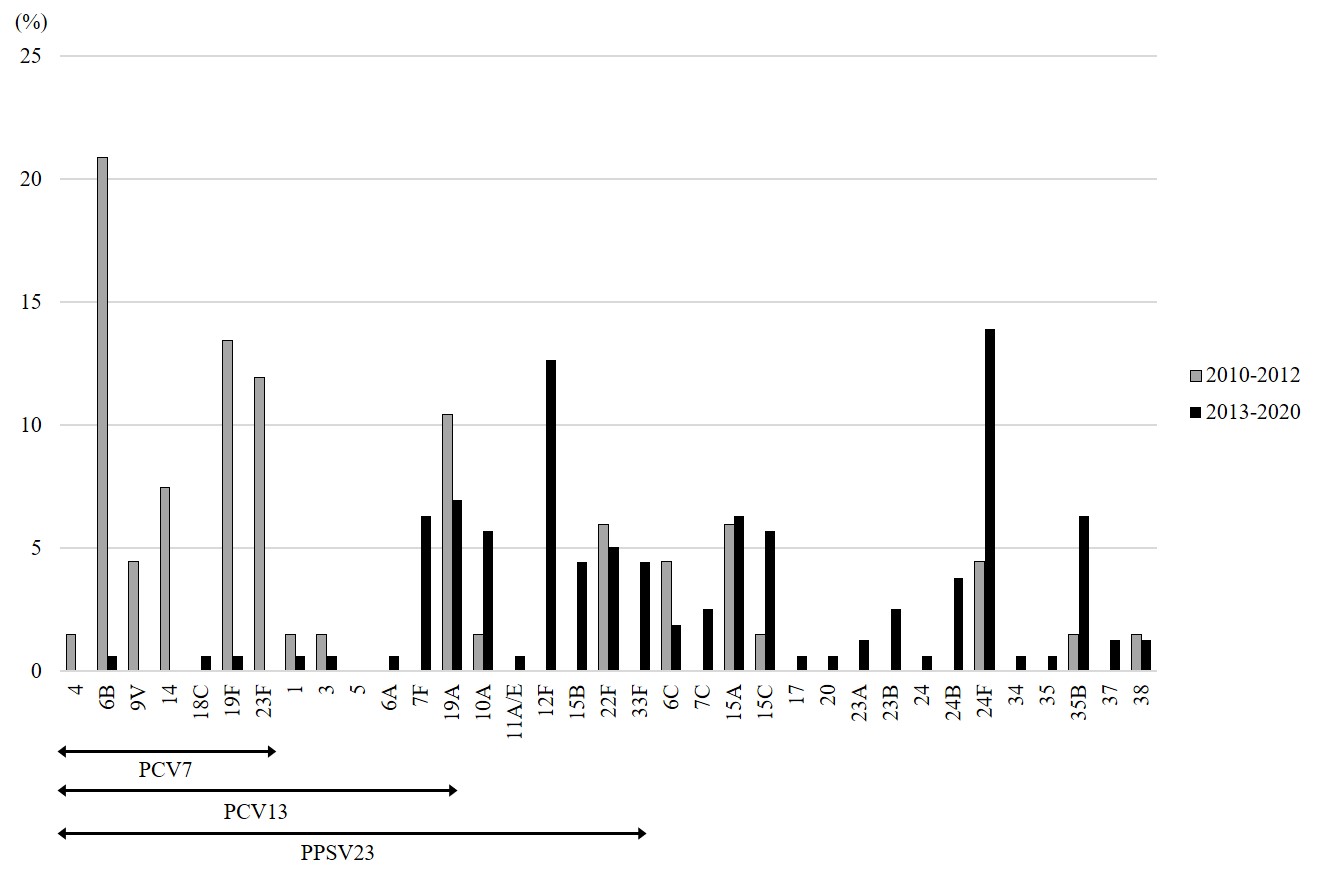

Supplement: Supplementary file 1 [file S0950268822000395sup001.jpg]
